# Similar additive effects of doxorubicin in combination with photon or proton irradiation in soft tissue sarcoma models

**DOI:** 10.3389/fonc.2023.1211984

**Published:** 2023-07-12

**Authors:** Teresa Bernardo, Carina Behrends, Diana Klein, Anna Kuntze, Beate Timmermann, Cläre von Neubeck

**Affiliations:** ^1^Department of Particle Therapy, University Hospital Essen, University of Duisburg-Essen, Essen, Germany; ^2^West German Proton Therapy Center Essen (WPE), Essen, Germany; ^3^West German Cancer Centre (WTZ), University Hospital Essen, Essen, Germany; ^4^Faculty of Physics, Technical University (TU) Dortmund University, Dortmund, Germany; ^5^Institute of Cell Biology (Cancer Research), University Hospital Essen, University of Duisburg-Essen, Essen, Germany; ^6^Gerhard Domagk Institute of Pathology, University Hospital Münster, Münster, Germany; ^7^German Cancer Consortium (DKTK), Essen, Germany

**Keywords:** soft tissue sarcoma, proton beam radiotherapy, combined treatment, doxorubicin, additive effect

## Abstract

High-precision radiotherapy with proton beams is frequently used in the management of aggressive soft tissue sarcoma (STS) and is often combined with doxorubicin (Dox), the first-line chemotherapy for STS. However, current treatment approaches continue to result in high local recurrence rates often occurring within the treatment field. This strongly indicates the need of optimized treatment protocols taking the vast heterogeneity of STS into account, thereby fostering personalized treatment approaches. Here, we used preclinical STS models to investigate the radiation response following photon (X) or proton (H) irradiation alone and in combination with different treatment schedules of Dox. As preclinical models, fibrosarcoma (HT-1080), undifferentiated pleiomorphic sarcoma (GCT), and embryonal rhabdomyosarcoma (RD) cell lines were used; the latter two are mutated for TP53. The cellular response regarding clonogenic survival, apoptosis, cell-cycle distribution, proliferation, viability, morphology, and motility was investigated. The different STS cell types revealed a dose-dependent radiation response with reduced survival, proliferation, viability, and motility whereas G2/M phase arrest as well as apoptosis were induced. RD cells showed the most radiosensitive phenotype; the linear quadratic model fit could not be applied. In combined treatment schedules, Dox showed the highest efficiency when applied after or before and after radiation; Dox treatment only before radiation was less efficient. GCT cells were the most chemoresistant cell line in this study most probably due to their TP53 mutation status. Interestingly, similar additive effects could be observed for X or H irradiation in combination with Dox treatment. However, the additive effects were determined more frequently for X than for H irradiation. Thus, further investigations are needed to specify alternative drug therapies that display superior efficacy when combined with H therapy.

## 1 Introduction

Sarcomas are a very rare disease with an incidence of 6 per 100.000 people representing 1%–2% of all adult and 12%–15% of all pediatric cancers (1). They originate from soft (mesenchymal) tissues (84%) or bones (14%) (2). Classification, including immunohistochemistry, is important in the context of diagnosis and therapeutic option (3–5). Currently, >70 histological subtypes with specific morphology have been identified so far (3). Rhabdomyosarcomas (RMS) are the most common soft tissue tumor (STS) in children accounting for >50% of the cases (6). Undifferentiated pleiomorphic sarcoma (UPS) including giant cell tumors (GCT) is the most common STS in late adulthood with a high rate of local recurrence and distal metastasis (7) and 5-year survival of patients of ca. 50% (4). Fibrosarcoma generally concerns all age groups, but subtypes vary significantly between adults and children, e.g., rarely metastasizing infantile to highly malignant adult-type fibrosarcoma with poor prognosis (7). Independent of histology, sarcomas are generally treated multimodally in expert reference centers since there is a high need for individualized treatment approaches (8). Whereas surgical resection of the tumor remains as a primary treatment option, high-precision neoadjuvant or adjuvant radiotherapy (RT) was shown to improve local control rates (9). In particular, proton beam therapy (PBT) is gaining importance as a treatment option for STS due to the advantageous dose distribution. In contrast to photon-based intensity-modulated radiotherapy, PBT can spare critical normal tissue structures such as the central nervous system or other organs better while delivering an iso-effective dose to the tumor volume (2). The effects of photon (X) and proton (H) beams can be compared for various biological endpoints via the relative biological effectiveness (RBE). The RBE sets the photon and H doses, which induce the same biological effect in relation. In clinical treatment planning, the RBE of H is considered to be a constant 1.1 (10). In contrast, a large heterogeneity in RBE of H was shown for various sarcoma cell lines *in vitro* (11). STS shows a poor response to systematic treatments (9), and first-line drugs are still classical chemotherapies such as doxorubicin (Dox, anthracycline), ifosfamide, and dacarbazine (both alkylating drugs). The survival benefit for STS patients with low predicted overall survival was confirmed for anthracycline-based chemotherapy (12). However, alternative regimes to improve outcomes of STS such as combined radiation and chemotherapy approaches remain challenging (13). Despite recent advances in newly approved drugs and radiotherapy modalities, the 5-year overall survival for large and high-grade tumors is still poor with rates below 50% (14). Thus, there is an urgent need to optimize treatment protocols for combined radiochemotherapies, particularly with PBT and standard chemotherapy in STS (11), and to investigate (potential) additive effects of combined therapies relative to the mono-radiotherapy (15). This study therefore characterizes the effects of H irradiation alone and compares the effect to X irradiations alone and in combination with Dox in preclinical STS models (fibrosarcoma, undifferentiated pleiomorphic sarcoma, rhabdomyosarcoma). Furthermore, the sequence of the combined treatments was altered by applying Dox only before, before and after, or only after irradiation to gain insights in the effect size of chemotherapy and radiation modalities.

## 2 Materials and methods

### 2.1 Cell culture

The HT-1080 (ATCC CCL-121, fibrosarcoma, RD (ATCC CCL-136, embryonal rhabdomyosarcoma), and GCT (ATCC TIB-233, undifferentiated pleomorphic sarcoma/giant cell tumor) cell lines were obtained from the American Type Culture Collection. HT-1080 cells were isolated from a 35-year-old man who did not receive treatment. The cells are TP53 wild type (16). RD cells were derived from biopsy specimens of a 7-year-old woman with pelvic RMS previously treated with cyclophosphamide and radiation. GCT cells were derived from the lung of a 29-year-old man. The TP53 gene was mutated in RD (homozygous (17) and GCT (two heterozygous) cells. All cell lines were grown in medium supplemented with 10% (v/v) fetal bovine serum and penicillin–streptomycin (100 U/ml). The HT-1080 and RD cell lines were grown in Dulbecco’s modified Eagle’s medium (Thermo Fisher scientific, Waltham, USA), which was supplemented with 1% sodium pyruvate (Sigma-Aldrich, St. Louis, USA) for HT-1080 cells. GCT cells were grown in McCoy’s (Thermo Fisher Scientific, Waltham, USA). Cells were maintained at 37°C and 5% CO_2_ in a humidified incubator.

### 2.2 Photon irradiation

Photon irradiation hereafter referred to as X was performed using an ISOVOLT 320 X-ray machine (Seifert–Pantak, East Haven, CT) at 320 kV, 10 mA with a 1.65-mm aluminum filter, and a distance around 50 cm to the object being irradiated (18).

### 2.3 Proton irradiation

Proton irradiation hereafter referred to as H was performed with an IBA Proteus PLUS proton therapy system (IBA PT, Louvain-la-Neuve, Belgium) at the West German Proton Therapy Centre Essen (WPE). A clinical pencil beam scanning line with an IBA PBS-dedicated nozzle was used. Several proton beams were energy and intensity modulated layered to form a spread-out Bragg peak (SOBP) consisting of five energy layers of 118.8 MeV up to 129.9 MeV. The proton beam range was compensated with a range shifting block water equivalent thickness (WET) = 74 mm, material: polymethyl methacrylate (PMMA)) and an additional solid water phantom (RW3 plates, type SP34 IBA Dosimetry, composition: 98% polystyrene + 2% TiO_2_) with a WET of 3.3 cm to irradiate the cells in the middle of the SOBP. Cells in multiwell plates were irradiated with a homogeneous field with absorbed physical doses of 1, 2, 4, 6, or 8 Gy (field sizes: 20 × 20 × 1 cm^3^). Multiwell plates were positioned laterally and centered with the sample surface in the isocenter on the treatment table and irradiated with a gantry angle of 0°.

### 2.4 Doxorubicin treatment

**Figure 1:**
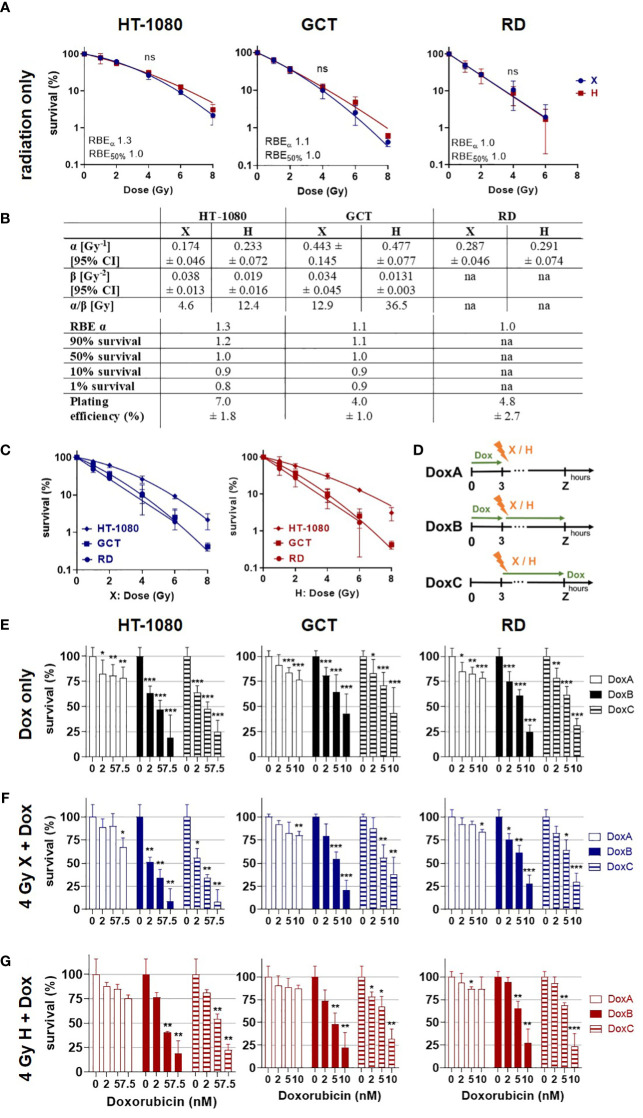
Colony formation assay. Clonogenic survival of HT-1080, GCT, and RD cells following **(A)** X radiation (blue) or H radiation (red) alone. HT-1080/GCT: fitted with the linear–quadratic model; RD: semi log line fit. **(B)** Table summarizing the fit parameter of the survival curves shown in **(A)**, the maximum RBEα, the RBE values to survival levels 90%, 50%, 10%, and 1%, and the plating efficiency of the cell models. RD cells were fitted with a semi log line fit, and no β-term was retrieved. **(C)** Cell survival curves following X (blue) or H (red) irradiation replotted from **(A)** to allow better evaluation of the radiation quality effects. **(D)** Summary of doxorubicin (Dox) treatment schedules. DoxA: 3 h before (mock) irradiation followed by media exchange without Dox, DoxB: 3 h before (mock) irradiation followed by media exchange containing Dox. Dox exposure until end of experiment. DoxC: (mock) irradiation followed by media exchange containing Dox. Dox exposure until end of experiment. Mock Dox treatments (medium without Dox) were performed for all conditions. **(E)** Dox treatment alone or in combination with **(F)** 4 Gy X irradiation or **(G)** 4 Gy H irradiation. Dox was applied according to **(D)**. Samples were normalized to matching 0 nM (+ irradiation) controls. n ≥ 3, statistical analysis: **(A)** paired t-test for whole curve comparing X vs. H. **(E-G)** Unpaired t-test comparing mono-/combined treatment vs. matching 0 nM control. p values > 0.05 (not significant, ns), < 0.05 (*), < 0.01 (**), and < 0.001 (***) were considered statistically significant.

The cytotoxic antibiotic doxorubicin (Dox) (2 mg/ml, Medac GmbH, Wedel, Germany) was purchased from and prepared by the pharmacy of the University Hospital Essen. For experiments, Dox was diluted in PBS (Invitrogen, Carlsbad, USA) and culture medium. Cells were treated in different sequences: 3 h before irradiation (DoxA), 3 h before irradiation and refreshed within 1 h after irradiation till the end of the experiment (DoxB), or 1 h after irradiation till the end of the experiment (DoxC) (**Figure 1D**).

### 2.5 Conditioned media

RD or GCT cells were cultured in normal growth media until confluence. The medium was collected, centrifuged, sterile-filtered (0.2 µm, Roth, Karlsruhe, Germany), and stored at −20°C until use. The conditioned medium was mixed with fresh medium as a 20% mixture for RD cells and a 40% mixture for GCT cells for the colony formation assay (19).

### 2.6 Colony formation assay

For the clonogenic survival, HT-1080 and GCT cells were preseeded 8 h and RD cells 24 h prior to radiation in triplicates in six-well plates. Cells were treated with Dox-containing culture medium. Following the irradiation, the media of all samples were changed with medium (HT-1080), conditioned medium (GCT and RD), or Dox-containing (conditioned) medium. The colonies were fixed after 9 (HT-1080), 10 (GCT), or 12 (RD) days depending on the cell doubling time (HT-1080: 24 h, GCT: 26 h, RD: 48 h), stained using 0.3% crystal violet dye (Roth, Karlsruhe, Germany) in 70% ethanol for 10 min at RT, rinsed with water, and air dried. Colonies with 50 cells were scored as surviving.

### 2.7 Flow cytometry analysis

Cells were plated 24 h before treatment in six-well plates. Propidium iodide (PI) staining and flow cytometry analysis for apoptotic DNA fragmentation (subG1 population) were performed 48, 72, or 96 h post treatment. Cells were incubated for 15–30 min at RT with a staining solution (0.1 M Tris, 0.1 M NaCl, 5 mM MgCl_2_, 0.05%, Triton X-100 (all Roth, Karlsruhe, Germany)), additional 62 µg/ml RNase A (AppliChem, Darmstadt, Germany), and 40 µg/ml PI (Sigma-Aldrich, St. Louis, USA) (20). Samples were analyzed by flow cytometry (FACSCalibur, Becton Dickinson, Heidelberg, Germany; FL-2) as described elsewhere (18). Cell-cycle phase distribution was analyzed with Kaluza software to identify the subG1 population (apoptotic DNA fragmentation, whole population), and in a second step, the living cell population (G1, S, G2/M phase) was investigated for a G2 arrest. Statistical analysis was performed in GraphPad Prism Version 8.3.0.

### 2.8 Migration assay

The migratory potential of cells was investigated with the migration assay 48 h post treatment at 0, 3, 6, 9, 24, and 48 h time points after scratch induction (**Supplementary Figure 1**). Wound closure was documented in images and determined by measuring the area of the scratch using ImageJ (Wayne Rasband, National Institutes of Health, US states) with the plugin Wound_healing_size_tool_updated (19). To calculate the maximum motility speed for each cell line, we calculated a simple linear regression between two time points (HT-1080: 0–3 h, GCT: 3–6 h, RD: 6–9 h) and determined the slope in the steep part of the curve. Additional morphological changes were evaluated by a sarcoma specialist on the basis of images of the migration assay.

### 2.9 Cell viability and proliferation analyses

The cell proliferation reagent WST-1 (in PBS 1:3, Roche, Rotkreuz, Schweiz) was used as a colorimetric assay for the quantification of cellular viability and cytotoxicity according to the manufacturer’s instruction (Roche, Rotkreuz, Schweiz). Optical densities were measured at 450 nm 60–90 min after incubation (BioTek Synergy H1 microplate reader, Agilent Technologies, Santa Clara, USA). Afterward, cells were fixed with glutaraldehyde (1% in PBS, Roth, Karlsruhe, Germany) for 15 min, stained with 0.5% crystal violet (CV) dye (Roth, Karlsruhe, Germany) in deionized water for 25 min, gently rinsed in water, and air dried overnight. The crystal violet dye was resolved in ECOSURF (0.2% in PBS, Roth, Karlsruhe, Germany) on a shaker for 20 min before optical density was measured at 540 nm (19). WST-CV data were normalized to 0 Gy or 0 nM controls.

### 2.10 Data analysis and statistics

Cell survival and dose response data were fitted using the linear quadratic equation:


$$SF=\;e^{-\left(\alpha D+\beta D^{2}\right)}$$

where SF denotes the surviving fraction of cells at dose D with curve fitting parameters α and β. Non-linear regression analysis was performed on survival curves using GraphPad Prism, version 8.3.0. RBE values for protons were calculated relative to 320 kV X-rays according to


$$RBE\;SF=\;\frac{D^{X\;SF}}{D^{H\;SF}}$$

where RBE SF is the RBE at a certain survival level (SF) and D^X SF^ and D^H SF^ are the X and H dose for an iso-effect, respectively.

Statistical analyses were performed with GraphPad Prism 8.3.0, and all data points represent at least three replicates with error bars representing the standard deviation (SD). All presented data were normalized to the experiment, time, and treatment matching controls. The SD for the controls of each assay was calculated as followed. For the colony formation assay (CFA), the plating efficiency (PE) was calculated. The corresponding SD represents the relative mean of the PEs. For subG1 levels (apoptosis) and the cell-cycle phase, the SD was calculated from the mean of relative subG1 or cell-cycle phase levels. For the cell viability and proliferation assay, measurements were normalized to 0 Gy control and the corresponding SD was calculated from the relative mean of measurements. For the migration assay, the SD was calculated from the mean of relative motility. The significant level was determined by unpaired (curve comparison) or paired t-test (data point comparison) with p values > 0.05 (not significant, ns),< 0.05 (*),< 0.01 (**), and< 0.001 (***) were considered statistically significant.

## 3 Results

### 3.1 Clonogenic survival: combined Dox treatment reduced clonogenic survival of STS cells more efficiently upon prolonged treatment

The CFA is the most reliable method to quantify clonogenic growth and survival following radiation as an important endpoint of the cellular response toward cytotoxic stimuli (21). In order to determine the radiation sensitivity of the different STS cells, CFA was performed following X or H irradiation (dose range 0–8 Gy). Plating efficiencies and survival curves were calculated from surviving colony numbers, and respective curves were fitted with the linear quadratic model (LQM) for HT-1080 and GCT cells and with a semi-log line for RD cells (**Figure 1A**). RD cells seem to be the most radiosensitive cell line, followed by GCT and HT-1080. Of note, no significant difference in survival curves between X- and H-irradiated STS cells could be estimated (**Figure 1A**). A cell line comparison of the response to X or H irradiation showed that RD, followed by GCT and HT-1080, was the most radiosensitive cells to both radiation modalities (**Figure 1C**). The RBEα defined as the ratio of α_H_/α_X_ shows for HT-1080 an elevated RBE of 1.3 indicating a higher sensitivity toward H irradiation (**Figure 1B**). This effect was not seen for GCT or RD cells. The RBE decreases with lower survival levels, which points toward a higher effectiveness for higher single X doses (≥6 Gy) relative to H irradiation in HT-1080 and GCT cells. Dox treatment at the indicated concentration (0–10 nM) alone was then used to determine respective chemosensitivities (**Figure 1E**). HT-1080 cells were most chemosensitive STS cells, and the maximum Dox concentration had to be reduced from 10 to 7.5 nM to archive surviving colonies. The longest Dox treatment, DoxB, reduced most effectively the cell survival in a dose-dependent manner (**Figure 1D**). In combination with X or H irradiation, the survival of all cell lines was even further reduced, again, with DoxB being most effective (**Figures 1F, G**). Of note, Dox treatment (only) before radiation (DoxA) was less effective than Dox after irradiation (DoxC) independent of cell model or radiation quality (**Figure 1E**). When comparing Dox treatment alone with combined treatment modalities, significant differences for HT-1080 (2 nM DoxB and X; 2 nM DoxB/C and H; 5 nM DoxC and X) and RD cells (2 nM DoxB/C and H) were revealed; GCT cells were not significantly affected. When comparing matching DoxA and DoxB or DoxC (alone or in combination with irradiation), significant differences for all Dox concentrations in HT-1080 and RD cells, for 5 nM and in GCT for 10 nM (**Figures 1E–G**) were evaluated.

### 3.2 Apoptosis: GCT cells are chemoresistant for Dox treatment alone independent of sequence but sensitive for combined treatment with radiation

**Figure 2:**
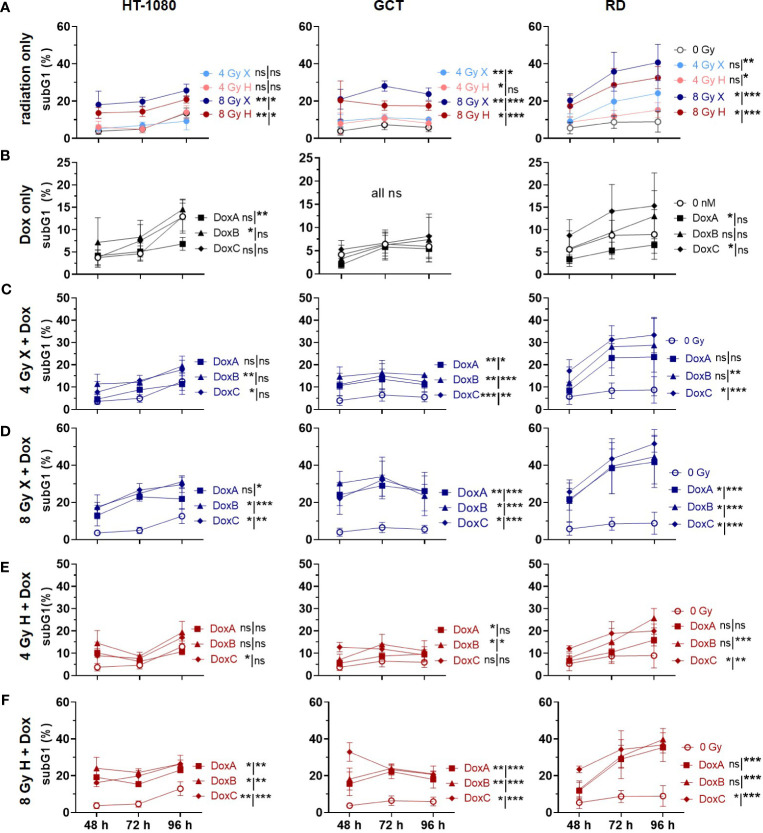
Flow cytometry comparing relative subG1 phase proportion (subG1) of whole-cell population (sub G1, G1, S, G2, M phase) of 0 Gy control and treatment of HT-1080, GCT, and RD cells following **(A)** radiation only with 4 and 8 Gy X (light/dark blue) or 4 and 8 Gy H (light/dark red). **(B)** Dox treatment with 10 nM DoxA (before), DoxB (before & after), or DoxC (after); Dox treatment schedule details in **Figure 1D**. **(C - F)** Combined treatment with DoxA, DoxB, or DoxC, and **(C)** 4 Gy X (blue), **(D)** 8 Gy X (blue), **(E)** 4 Gy H (red), or **(F)** 8 Gy H (red). n ≥ 3, statistical analysis: paired t-test for whole curve or unpaired t-test for 96 h timepoint (shown as whole curve | 96 h) comparing treatment vs. 0 Gy control. p values > 0.05 (not significant, ns), < 0.05 (*), < 0.01 (**), and < 0.001 (***) were considered statistically significant.

Apoptosis is a further mechanism of cell death following radiation exposure and the main mechanism of action for the DNA damaging drug Dox (22). According to the clonogenic survival measurements performed above, apoptosis induction was analyzed next within the first 96 h following X or H irradiation and 10 nM Dox treatment by determining apoptotic DNA fragmentation using flow cytometry analysis in combination with PI staining. Relative to controls (0 Gy, 0 nM Dox), the subG1 population increased with radiation dose and time after treatment in HT-1080 and RD cells whereas in GCT cells only a radiation dose-dependent effect was seen (**Figure 2A**). Dox treatment alone had minor effects in HT-1080 and RD cells and did not affect GCT cells (**Figure 2B**). Combined X or H irradiation with Dox showed a radiation dose-dependent higher apoptosis rate and a Dox schedule-dependent difference with DoxB and DoxC being more effective than DoxA (**Figures 2C–F**). Matching X and H samples were compared by identifying the potential influence of the radiation quality (**Supplementary Figure 2**). Only the apoptosis rates in HT-1080 were statistically different following 8-Gy radiation alone (**Supplementary Figure 2A**). However, GCT and RD cells are shown in combination with DoxA and RD cells also in combination with DoxB significant differences following 8 Gy (**Supplementary Figures 2B, C**). The cellular response following DoxC was radiation quality independent (**Supplementary Figure 2D**). The data were normalized to the respective dose (4 or 8 Gy), radiation quality (X or H), and time matching (48, 72, 96 h) of samples to identify potential additive or synergistic effects in combined treated samples (**Supplementary Figure 3**). For HT-1080 cells, an additive effect could be identified for both irradiation qualities but only for DoxB and DoxC. In contrast, in GCT cells, no additional effect was seen for H irradiation and any Dox treatment. RD cells showed additive effects for X and H irradiation with DoxC (**Supplementary Figure 3**).

### 3.3 Cell cycle distribution: accumulation of the G2/M population in GCT and RD cells following treatment with irradiation or Dox

**Figure 3:**
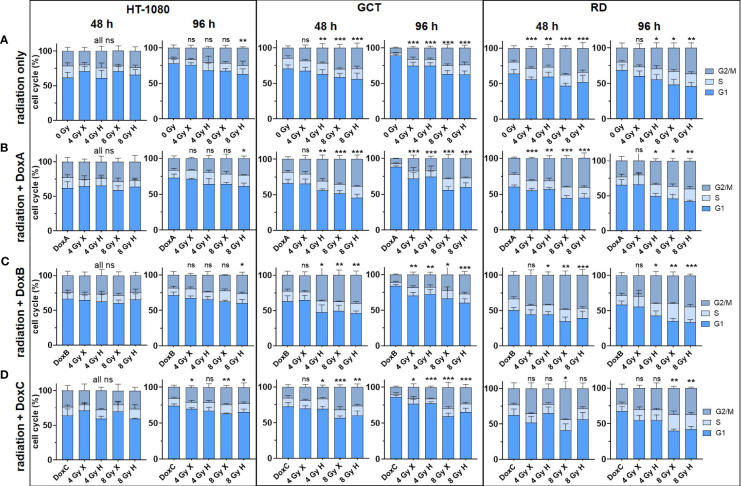
Flow cytometry comparing the relative cell-cycle phase (G1 + S + G2/M = 100%) of HT-1080, GCT, and RD cells at 48 and 96 h following **(A)** 4 and 8 Gy X or H radiation only. **(B–D)** Combined treatment with radiation and **(B)** DoxA (before), **(C)** DoxB (before and after), and **(D)** DoxC (after). Dox treatment schedule details in **Figure 1D**. n ≥ 3, statistical analysis: unpaired t-test for each timepoint comparing treatment vs. matching control of the G2 phase. p values > 0.05 (not significant, ns), < 0.05 (*), < 0.01 (**), and < 0.001 (***) were considered statistically significant.

DNA damaging treatment such as radiation and chemotherapy can induce a transient or permanent cell-cycle arrest stopping the proliferation of damaged cells and providing an opportunity for repair (23). Therefore, the effect of mono- or combined treatment with Dox and X or H radiation on cell cycle phases was analyzed (**Figure 3**). The HT-1080 cell did not show a cell-cycle alteration within 96 h after the indicated treatments. In contrast, GCT and RD cells accumulated in the G2/M phase 48 h after treatment with radiation only, or in combination with DoxA and DoxB. For the most intense treatment (DoxB 8 Gy X or H), 37.7% and 40.2% for GCT and 48.6% and 47.3% for RD cells accumulated in the G2/M phase at 48 h, respectively. Arrests were beginning to resolve at 96 h post treatment; significant changes relative to controls could still be detected (**Figure 3**).

### 3.4 Proliferation: prolonged Dox treatment combined with irradiation reduced proliferation activity of STS cells

**Figure 4:**
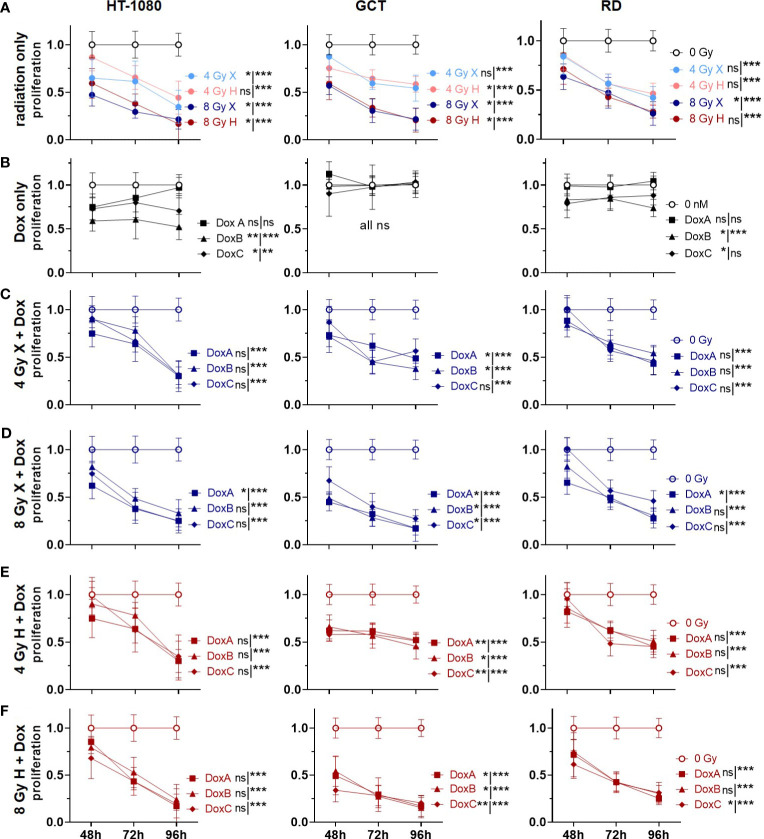
CV assay comparing the relative number of proliferating cells (proliferation; normalized to matched control) of 0 Gy control vs. treatment of HT-1080, GCT, and RD cells following **(A)** radiation only with 4 and 8 Gy X (light/dark blue) or 4 and 8 Gy H radiation (light/dark red). **(B)** Dox treatment with 10 nM DoxA (before), DoxB (before and after), or DoxC (after). Dox treatment schedule details in **Figure 1D** **(C–F)** Combined treatment with DoxA, DoxB, or DoxC and **(C)** 4 Gy X (blue), **(D)** 8 Gy X (blue), **(E)** 4 Gy H (red), and **(F)** 8 Gy H (red). n ≥ 3, statistical analysis: paired t-test for whole curve or unpaired t-test for the 96 h timepoint (shown as whole curve | 96 h) comparing treatment vs. 0 Gy control. p values > 0.05 (not significant, ns), < 0.05 (*), < 0.01 (**), and < 0.001 (***) were considered statistically significant.

Due to the G2/M phase arrest in two cell lines (GCT and RD cells), we hypothesized that radiation might also reduce the general proliferation activity. Cellular proliferation levels following irradiation and Dox treatment were then estimated for the different STS cells using the crystal violet assay (**Figure 4**). Relative to controls (0 Gy, 0 nM Dox), all cell lines showed reduced proliferation activities following both radiation qualities in a dose-dependent manner (**Figure 4A**). Dox treatment alone—in either treatment schedule—exhibited effects on the proliferation levels of GCT cells. DoxB and DoxC treatment schedules in contrast were able to reduce proliferations in HT1080 and RD cells (**Figure 4B**). When using radiation treatment in addition, all combinatory treatments significantly lowered proliferation activities of all STS cell lines investigated 96 h post onset of treatment (**Figures 4C-F**). Time- and dose-matching X- and H-exposed samples were additionally compared by identifying the potential influence of the radiation quality (**Supplementary Figure 4**). No significant changes could be found with the exemption of whole curve comparison of HT-1018 cells following 4 Gy and DoxA (**Supplementary Figure 4B**). To identify potential additive or synergistic effects in combined treated samples, the data were normalized to the respective dose (4 or 8 Gy), radiation quality (X or H), and time matching (48, 72, or 96 h) samples (**Supplementary Figure 5**). GCT cells were the most affected cell line, and additive effects were found for all Dox conditions with X irradiation (**Supplementary Figures 5A, B**). Following H irradiation, much fewer effects could be detected. In contrast, RD cells were the least affected cell line (**Supplementary Figures 5B, C**). However, DoxB seems to be the most efficient for all cell lines (**Supplementary Figures 5**).

### 3.5 Cell viability: additive effects could be identified for prolonged Dox treatment and X but not for H

**Figure 5:**
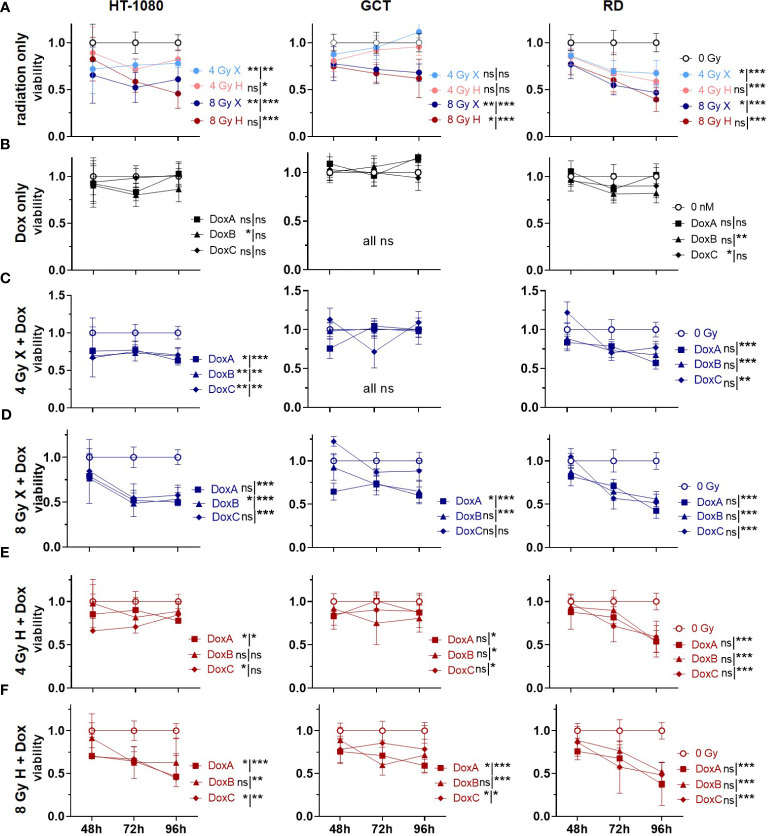
WST assay comparing relative number of viable cells (viability; normalized to matched controls) of 0 Gy control vs. treatment of HT-1080, GCT, and RD cells following **(A)** radiation only with 4 and 8 Gy X (light/dark blue) or 4 and 8 Gy H radiation (light/dark red). **(B)** Dox treatment only with 10 nM DoxA (before), DoxB (before and after), or DoxC (after). Dox treatment schedule details in **Figure 1D** **(C–F)** Combined treatment with DoxA, DoxB, or DoxC and **(C)** 4 Gy X (blue), **(D)** 8 Gy X (blue), **(E)** 4 Gy H (red), and **(F)** 8 Gy H (red). n ≥ 3, statistical analysis: paired t-test for whole curve or unpaired t-test for 96 h timepoint shown as (whole curve96 h) comparing treatment vs. 0 Gy control. p values > 0.05 (not significant, ns), < 0.05 (*), < 0.01 (**), and < 0.001 (***) were considered statistically significant.

Cellular viabilities were measured via metabolic activities following combined treatment of X or H irradiation with Dox using the WST-1 reagent (**Figure 5**). Relative to controls (0 Gy, 0 nM Dox), both radiation qualities lowered the cell viability in a dose-dependent manner. GCT recovered independent of radiation quality to the control level after 4 Gy and 96 h, whereas HT-1080 and RD did not (**Figure 5A**). Dox alone had only minor effects on cellular viabilities; GCT cells were not affected, whereas minor effects of DoxB (HT-1080 cells) and DoxC (HT-1080, RD cells) were seen (**Figure 5B**). The combination of X and all Dox treatments significantly reduced the cell viability in HT-1080 and RD cells 96 h post treatment; in GCT cells, only 8 Gy X and DoxA and DoxB was effective (**Figures 5C-D**). To 96 h post treatment, H and Dox significantly decreased metabolic activity in all cell lines and treatments except HT-1080 to 4 Gy DoxB and DoxC (**Figures 5E-F**). Time- and dose-matching X- and H-irradiated samples were assessed to identify the potential influence of the radiation quality (**Supplementary Figure 6**). Again, no significant difference between X- and H-irradiated samples 96 h post treatment could be found, exempt GCT to 4 Gy (96 h) or 8 Gy (whole curve). In contrast, whole curve comparisons showed significant changes for HT-1080 and RD following irradiation and DoxA or DoxB. (**Supplementary Figures 6B, C**). To further reveal potential additive effects in combined treated samples, data were normalized to the respective dose (4 or 8 Gy), radiation quality (X or H), and time matching (48, 72, 96 h) samples (**Supplementary Figure 7**). For X-exposed samples, additive effects were found for HT-1080 and RD cells whereas GCT cells were not affected (**Supplementary Figures 7A, B**). No additive effects were identified following H irradiation and any Dox treatment (**Supplementary Figures 7C, D**).

### 3.6 Cell morphology analysis post treatment: radiation effects morphology more pronounced than Dox

**Figure 6:**
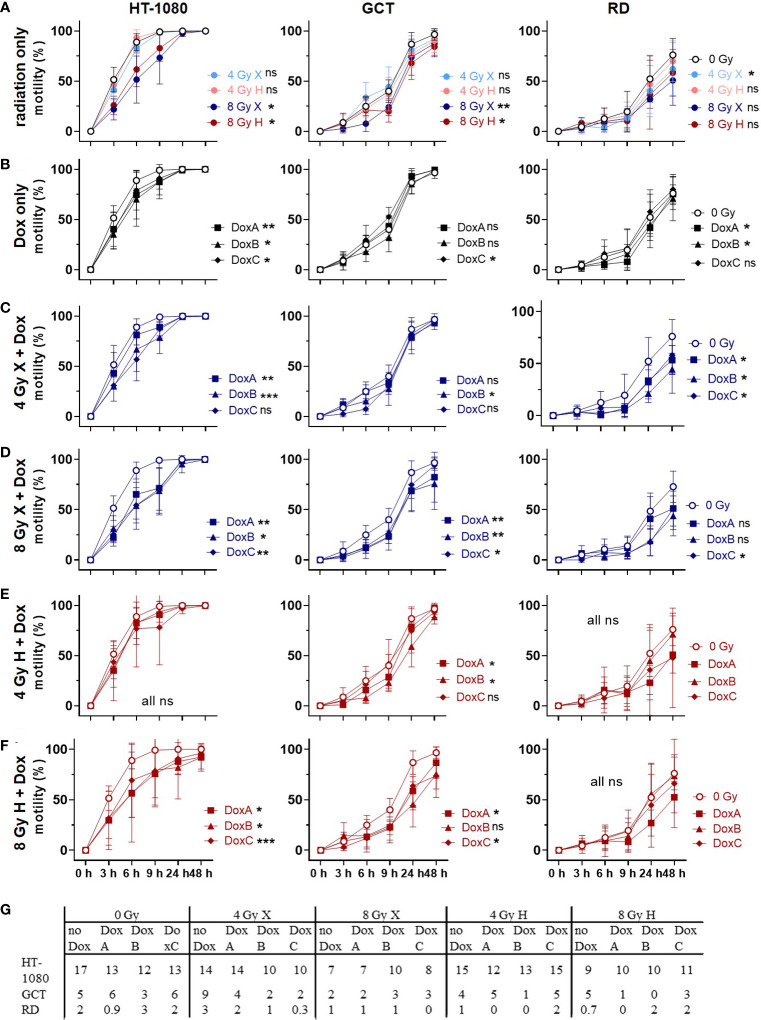
Migration assay comparing motility of 0 Gy control vs. treatment of HT-1080, GCT, and RD cells following **(A)** radiation only with 4 and 8 Gy X (light/dark blue) or 4 and 8 Gy H (light/dark red) radiation. **(B)** Dox treatment with10 nM DoxA (before), DoxB (before and after), or DoxC (after). Dox treatment schedule details in **Figure 1D** **(C–F)** Combined treatment with DoxA, DoxB, or DoxC and **(C)** 4 Gy X (blue), **(D)** 8 Gy X (blue), **(E)** 4 Gy H (red), and **(F)** 8 Gy H (red). **(G)** Maximum of migration speed for each cell line extracted from the exponential phase of the curve via linear regression. n ≥ 3, statistical analysis: paired t-test for whole curve comparison: treatment vs. 0 Gy control for HT-1080 cells until the scratch was closed (0–9 h) and for GCT and RD cells over the whole observation period of 0–48 h. p values > 0.05 (not significant, ns), < 0.05 (*), < 0.01 (**), and < 0.001 (***) were considered statistically significant.

The migration assay was used to study morphological changes upon treatment (**Figure 6**, **Supplementary Figure 1**). Untreated HT-1080 cells, under the given cell culture conditions, appear small, with a spindled to round shape, with aspects of a whirling architecture. The nuclei are hyperchromatic and broadly isomorphic. Upon X and H irradiation, HT-1080 cells seem slightly enlarged and appear predominantly in spindle shape with long cytoplasmatic processes; nuclei appear to be increasingly anisomorphic. No morphological changes were seen following DoxA alone or in combination with any radiation treatment, compared with X or H irradiation alone. DoxB and DoxC, however, induced spindle-shaped cells with long cytoplasmic processes and anisonucleosis. Combined treatment with DoxB or C and both radiation qualities increased the amount of anisonucleosis and increased the frequency of cells, which lost their cytoplasmic processes and their bipolar spindled shape.

Unirradiated GCT cells show a largely homogeneous spindled morphology with long cytoplasmic processes creating intercellular connections. Following X and H irradiation, cells show cytoplasmic and nuclear enlargement; furthermore, multinucleated cells appear. Few GCT cells develop a dendritic shape with fibroblastic appearance. Monotreatment with DoxA or combined treatment with any radiation and DoxA did not alter the morphology. Cells under DoxB or DoxC treatment alone appear with extended cytoplasmatic processes in GCT cells. The morphological effects upon irradiation and chemotherapy alone were also seen following combined treatment with irradiation and DoxB or DoxC.

Untreated RD cells present as networking spindled cells with long cytoplasmic processes and broadly isomorphic nuclei. In co-localization, few single polygonal cells with larger, roundish nuclei are apparent. Upon X and H irradiation, cells and nuclei appear enlarged and increasingly anisomorphic, and multinucleated cells show up. The cytoplasm becomes granular, and some cells loose the spindled morphology. Independent of treatment schedule, Dox treatment alone had no effect on the morphology of RD cells, like combined therapy with DoxA and radiation. However, exposure to combined X or H radiation and DoxB or C treatment led to the appearance of long thin processes, fibroblast-like and dendrite-like cell shapes, and increasing anisonucleosis. Overall, irradiation effects morphology of STS cells more pronounced than Dox treatment.

### 3.7 Cell motility: irradiation and Dox treatment reduced motility, but X-Dox was more effective than H-Dox

Finally, the migration assay was used to study cellular motilities by measuring the surface area that cells occupy over time after treatment with X or H irradiation and 10 nM Dox (**Figure 6**, **Supplementary Figure 1**). Relative to controls (0 Gy, 0 nM Dox), motilities for HT-1080 and GCT were significantly reduced following 8 Gy irradiation. However, RD cells lowered the motility only after 4 Gy X significantly (**Figure 6A**). Dox treatment lowered the motility in a cell line-dependent manner with HT-1080 being the most and GCT being the least affected (**Figure 6B**). Combined treatment of X irradiation and Dox reduced the motility in all cell lines where the 8 Gy dose was again more effective in HT-1080 and GCT, whereas RD was more affected after 4 Gy and Dox (**Figures 6C, D**). Interestingly, the combined treatment of Dox and H irradiation had less effects on cellular motilities. Only HT-1080 (8 Gy only) and GCT (both doses) cells showed significant effects (**Figures 6E, F**). The maximum speed of cell migration was calculated to form the exponential phase of the motility curves (HT-1080 cells 0–3 h; GCT cells: 3–6 h and RD cells 6–9 h). With the exemption of 4 Gy X in GCT and RD cells, the cell motility was reduced by radiation, all Dox schedules, and combined treatments relative to controls (0 Gy, 0 nM Dox, **Figure 6G**). The data were additionally normalized to the respective dose (4 or 8 Gy), radiation quality (X or H) (**Supplementary Figure 8**). Time- and dose-matching X and H irradiated samples were assessed to identify the potential influence of the radiation quality (**Supplementary Figure 9**). With the exemption of 4 Gy with DoxB in HT-1080 and 8 Gy with DoxC in RD, no significant influence of the radiation quality on the motility of the cells could be measured.

## 4 Discussion

In clinical practice, the established chemotherapy protocols of X-based radiochemotherapy (24) are adopted for H-based radiochemotherapy (8). Unfortunately, there is a lack of large clinical trials investigating the effects of combined H-based radiochemotherapy (25). In order to increase the body of preclinical data to optimize and improve established treatment protocols for combining radiotherapy, particularly with H and standard chemotherapy in STS, the effects of H irradiation compared with X irradiation and combined with the chemotherapeutic drug Dox in three different sequences in three STS models were evaluated.

In this study, the clonogenic cell survival, apoptosis induction, cell-cycle effects, proliferation, viability, morphological changes and cellular motility were investigated. It is shown that HT-1080 were the most radioresistant and RD the most radiosensitive cell lines (**Figure 1C**). GCT cells were most resistant to Dox treatment. For all cell lines, the longest Dox treatment (DoxB) showed the highest effectiveness (**Figures 1E–G**). The DoxC schedule reflects the treatment situation in the clinics where patients with low predicted overall survival benefit from adjuvant chemotherapy (12). DoxB and DoxC are superior to DoxA (**Figures 1E-G**), supporting the importance of the Dox treatment after radiation. Overall, the colony formation assay is the most relevant assay for the clinics because it investigates the long-term survival of STS cells. For all combined treatment scenarios, additive effects could be found. Overall, the combination of Dox and X seems to be more effective than Dox and H (**Figures 1F, G**).

The different STS cell types investigated (HT-1080 fibrosarcoma, GCT undifferentiated pleiomorphic sarcoma, and RD embryonal rhabdomyosarcoma cells) revealed a dose-dependent RT response with RD cells exhibiting the most radiosensitive phenotype followed by GCT cells and the quite radioresistant HT-1080 STS cells (**Figure 1**). Of note, no superior effects could be estimated for H versus X irradiation. Presented experiments were performed in the middle of the irradiation field (center of the SOBP), as this represented the predominant situation in the irradiation field of H therapy (26). However, the cell survival curves were not significantly different for all the investigated models (**Figure 1A**) and the determined RBEs were in the range of the clinical assumption of 1.1 (27). RBE values as low as 0.8 were found for survival level 1% (**Figure 1B**), which is indicative of a higher effectiveness of X radiation. Other groups have found increased RBE values representing a higher biological effectiveness of H irradiation in the distal fall-off of the Bragg peak (11). For other entities such as brain tumors, it has been discussed that the increased RBE at the end of the proton range can lead to increased side effects in healthy tissue (11). The clinical evidence for these effects remains weak (28). Therefore, future experiments should investigate the cellular response in this region of the treatment field. Striking was the linear curve progression of the RD cells. This indicates a decreased DNA damage repair capacity (29) of the cells and a high sensitivity to radiotherapy. In follow-up studies, the functional mutational status of DNA repair proteins should be clarified for this cell line. The α/β ratios of HT-1080 and GCT cells are also of interest (**Figure 1B**). Here, the ratios for H irradiation are higher in both cases. This could be an indication of reduced fractionation sensitivity of the cells (30).

Concerning the chemosensitivity of investigated STS cells, a pronounced chemotherapy sensitivity was estimated for each cell line. Significant differences between Dox mono treatment and Dox radiation were found for HT-1080 and RD cells. However, survival of GCT cells was not significantly altered in combined treatment relative to Dox monotherapy. When comparing the different Dox schedules, DoxB and DoxC were superior to DoxA. Dox and ifosfamide remain the most effective chemotherapy drugs available for STS tumors (31). However, management of STS is increasingly subtype-dependent and resistance for Dox is present. Resistance mediating molecular alterations such as the mutation of TP53 was discussed since p53-dependent apoptosis is the main mechanism of action of Dox (32).Unfortunately, the investigation of new molecular targets only showed an incremental progress and no superior effect relative to Dox (13). Nevertheless, patients with undifferentiated pleomorphic sarcoma (UPS, GCT cells) showed the highest overall response from treatment with monoclonal antibodies against PD-L1 (33). TP53 mutations are mostly associated with increased aggressiveness and radio resistance (34). The sarcoma cells studied here all showed to be positive for apoptotic cell death (**Figure 2**). However, apoptosis induction was significantly increased after radiation treatment compared with apoptosis after chemotherapy alone (**Figures 2A, B**). Mutations in the TP53 gene are known for the RD (homozygous mutation of TP53 (17)) and GCT (two heterozygous TP53 mutations (ATCC)) cell lines, whereas HT-1080 is proficient for TP53 (35). Especially in GCT cells, no significantly increased apoptosis rates could be measured after treatment with Dox alone or in combination with H irradiation (**Figures 2B–F**). Nevertheless, it seems that the sequence of treatment has an impact on apoptosis rate and exclusive Dox treatment before irradiation is less effective than (before and) after radiation treatment (**Figure 2B**). Clinically, Dox chemotherapy is given as adjuvant or neoadjuvant intervention relative to radiotherapy. It is administered as a bolus injection within a few minutes or as a continuous intravenous infusion over several hours to days (36). The blood clearance of Dox varies widely inter-individually but extends over several days (36) so it can be assumed that Dox is present in sufficient amounts in tumor cells at the time of irradiation. All Dox experiments were performed in three different sequences with Dox treatment 3 h before irradiation (DoxA), 3 h before and refreshment within 1 h after irradiation (DoxB), or only within 1 h after irradiation (DoxC) (**Figure 1D**). Prolonged treatment with Dox in schedule DoxB or DoxC showed that major effects especially in combination with radiation additive effects could be determined (**Figures 2B-F**).

The mutation of RD and GCT cells for the TP53 gene is also reflected in the lack of p53-mediated G1/S cell-cycle arrest (37). Therefore, the cells temporarily arrest in the G2/M phase to repair DNA damage (38). In subsequent studies, the distribution and kinetics of DNA repair proteins might gain insight into the repair pathways used after X and H irradiation. First evaluations of decisive DNA repair protein levels however seemed to be unaltered in the STS cell lines investigated, at least under non-radiating conditions (data not shown). The increased repair of DNA damage via homologous recombination is intensively discussed in the context of H irradiation (38) and could be a starting point for the development of alternative drug therapy for STS.

Corresponding effects are demonstrated for cellular proliferations and viabilities after treatment (**Figures 4**, **5**): All cell lines showed decreased proliferation activities and viabilities after irradiation (**Figures 4A**, **5A**). For the Dox treatment alone, no effect could be determined in GCT cells for either endpoint, whereas effects for HT-1080 and RD cells were detected (**Figures 4B**, **5B**). To investigate the additive effect of combined treatment in comparison with the mono-treatment with radiation (15), the data were normalized to matching controls (**Supplementary Figures 5, 7**). However, additive effects were determined for GCT cells on proliferation especially after combination with Dox and X, whereas the endpoint viability was not additively affected. These additive effects belong to the *in vitro* synergy, which differs from the therapeutic synergy (39).Taken together, these data suggest that GCT cells have to some extent a resistance to Dox and are most inactivated by irradiation. No particular sensitivity to a beam quality could be determined (**Supplementary Figures 4, 6**). In contrast, HT-1080 and RD cells are sensitive especially to prolonged Dox treatment and the combination with radiation, whether X or H, shows additive effects (**Supplementary Figures 5**, **7**). Another important aspect is the different response of the cells in the cell viability and proliferation assay. While the proliferation of the cells is much more reduced following treatment (**Figure 4A**), the cell viability often recovers until 96 h (**Figure 5A**). Additionally, in the CFA with HT-1080 cells, single cells and colonies with less than 50 cells could be detected even after high-dose irradiation with 8 Gy, indicating that these cells are mitotically dead and stopped proliferating but are metabolically still active (40).

In all cell lines, radiation induced more changes in cell morphology compared with Dox treatment. However, no distinct differences in morphology between the radiation qualities (X or H) could be detected. Future work should include more time points, radiation doses, and additional treatment with relevant particles like carbon or oxygen ions to investigate the LET or RBE effect on morphology (41). Treatment-induced loss of bipolarity, prolonged cytoplasmic processes, and cell-shape alterations were seen for all cell lines indicating cell damage and cellular plasticity. For sarcoma, the transition from mesenchymal to (partial) epithelial (M(p)ET) cell type has been described and discussed as a potential biomarker for tumor treatment response (42). MET and the reversed-process epithelial to mesenchymal transition (EMT) have been discussed to contribute to doxorubicin resistance (43). Upregulation of EMT/MET genes has been reported, e.g., in rhabdomyosarcomas (44), which could be used for the development of new targeted drugs (45). The here found resistance for Dox of GCT cells corresponds with some gene analyses where genes, which are involved in chemoresistance (e.g., RAB22a and S100P), were upregulated in UPS. Furthermore, an upregulation of EMT-related genes and a downregulation of epithelial markers are common in UPS. The development of new drugs is ongoing. One example is eribulin, a novel microtubule inhibitor (45). Additionally, in context to rhabdomyosarcoma cases, upregulations of CDH1 (epithelial marker), SLUG (inducer of EMT), and MMP9 (matrix-modifying enzyme) are reported (44). In our study, no clear indications for MET or EMT could be seen based on cell cultures. To further analyze the potential cellular plasticity upon mono- or combined treatment, additional biomarker stainings for MET, e.g., N-cadherin, vimentin, and fibronectin, or EMT, e.g., E-cadherin, occludins, and claudins, are needed (46). To confirm the *in vitro* results, further investigations should be performed on tumor sections from *in vivo* or *in ovo* experiments. In our study, GCT and RD cells showed a pronounced resistance to Dox treatment of any schedule, which can in part be explained by the mutated TP53 gene. In primary STS cultures, a high mutation rate in apoptotic signaling genes (TP53, ATM, PIK3CB, PIK3R1, NTRK1, CSF2RB) was found and linked to Dox resistance (32). Future experiments should include molecular analysis regarding apoptosis and migration biomarkers to understand the additive effects mediated by anthracycline-based regimen. The involved genes and pathways could serve as new targets for personalized treatment approaches in sarcoma patients.

Finally, the migratory capacity of the three STS lines was investigated following the different radiation modality treatment with or without the different combined Dox schedules (**Figure 6**). For conventional X radiotherapy, an increased cell motility was shown, which holds the potential to promote invasion and metastasis (47). For the treatment of sarcoma, H radiotherapy is gaining importance (2). For example, for Ewing sarcoma cells (48), as well as for other cancer entities, e.g., for breast cancer cells (49), the enhanced motility following Dox treatment or X irradiation was already shown, but there is a lack of data for STS in general. Analysis of the motility in the three STS lines here revealed reduced migratory capacities following Dox and H treatment (**Figures 6E, F**). In addition to apoptosis, the damage of the cellular membrane, which may influence the motility as well, is a further mechanism of action of Dox (22). Conclusively, the improved action of combined radiochemotherapy as investigated here not only improved the therapeutic response concerning cell survival but even reduced the migration/invasion potential especially following combined treatment with a prolonged sequence (DoxB or DoxC) (**Figures 6C-F**).

In summary, no clear advantage of H therapy over X therapy could be revealed in preclinical STS models. Experiments were performed in the center of the SOBP and not at the distal fall-off, where enhanced RBE values are described (10). RD rhabdomyosarcoma cells are quite radiosensitive followed by GCT undifferentiated pleiomorphic sarcoma cells. HT-1080 fibrosarcoma cells showed the highest radioresistance while being sensitive to Dox treatments due to wt TP53 (50). For the cell models used, prolonged Dox treatment was revealed as most effective. Combination of H radiations with Dox showed for most endpoints similar effects compared with X irradiation. Currently, the measured effects can be labeled as “cell line specific”. To translate our findings to “STS subtype specific”, more experiments with cells of the respective histology needs to be performed. Subtype-specific treatment approaches of STS increased constantly (13). A recent review summarized all published and publicly available STS cell lines and found only 45 histological subtypes represented in cell lines whereas 133 subtypes were not. For the here used histological subtypes, alternative cell models are available in sufficient numbers for fibrosarcoma and rhabdomyosarcoma, but not for undifferentiated pleiomorphic sarcoma/giant tumor cells (3). Conclusively, the presented findings strongly suggest that alternative drug therapies should be developed for combination therapy with H. The ultimate goal would be an individualized drug treatment tailored to the patient in combination with high-precision radiotherapy after (partial) surgical removal of the tumor.

## Data availability statement

The original contributions presented in the study are included in the article/**Supplementary Material**. Further inquiries can be directed to the corresponding author.

## Author contributions

TB and CN conceptualized the study. Data acquisition was performed by TB, CB, and AK. CB calculated the proton beam fields for cell irradiation and AK analyzed the morphological changes following treatment. Analysis and interpretation of data were executed by TB, CN, and AK. Visualization was done by TB. DK provided protocols for proliferation, viability, and motility assay and provided temporary project supervision. The first draft of the manuscript was written by TB. CN and AK wrote parts of the manuscript. BT made proton beam time available. All authors revised the manuscript and approved the final version.

## Supplementary material

Supplementary Figure 1 Exemplary images of migration assay with **(A)** HT1080 cells, **(B)** GCT cells, and **(C)** RD cells following 8 Gy X or H radiation and combined treatment with DoxB (before & after; details see ) at different timepoints post scratch (0 h, 9 h, 48 h). 4 x magnification and scale bar = 553.3 µm.

Supplementary Figure 2 Flow cytometry comparing relative subG1 phase X vs. H of HT-1080, GCT, and RD cells following **(A)** radiation only with 4 and 8 Gy X (light/dark blue) or 4 and 8 Gy H (light/dark red) radiation. **(B-D)** combined radiation treatment with **(B)** DoxA (before), **(C)** DoxB (before & after), and **(D)** DoxC (after). Dox treatment schedule details in . n ≥ 3, statistical analysis: paired t-test for whole curve or unpaired t-test for 96 h timepoint shown as (whole curve| 96 h) comparing matching X vs. H.

Supplementary Figure 3 Flow cytometry analysis of additive effects of combined treatment compared with monotreatment (radiation only) of relative subG1 phase (subG1) of HT-1080, GCT, and RD cells following combined treatment with 10 nM DoxA (before), DoxB (before & after), or DoxC (after) and **(A)** 4 Gy X (blue), **(B)** 8 Gy X (blue), **(C)** 4 Gy H (red), **(D)** 8 Gy H (red). Dox treatment schedule details in . n ≥ 3, statistical analysis: paired t-test for whole curve or unpaired t-test for 96 h timepoint shown as (whole curve| 96 h) comparing monotreatment vs. combined treatment.

Supplementary Figure 4 CV assay comparing relative number of proliferating cells (proliferation; normalized to matched control) X vs. H of HT-1080, GCT, and RD cells following **(A)** radiation only with 4 and 8 Gy X (light/dark blue) or 4 and 8 Gy H (light/dark red) radiation. **(B-D)** combined radiation treatment with **(B)** DoxA (before), **(C)** DoxB (before & after), and **(D)** DoxC (after). Dox treatment schedule details in . n ≥ 3, statistical analysis: paired t-test for whole curve or unpaired t-test for 96 h timepoint shown as (whole curve| 96 h) comparing matching X vs. H.

Supplementary Figure 5 CV assay analysing the additive effects of combined treatment compared with monotreatment (radiation only) of relative number of proliferating cells (proliferation; normalized to matched control) of HT-1080, GCT, and RD cells following combined treatment with 10 nM DoxA (before), DoxB (before & after) or DoxC (after) and **(A)** 4 Gy X (blue), **(B)** 8 Gy X (blue), **(C)** 4 Gy H (red), and **(D)** 8 Gy H.(red). Dox treatment schedule details in . n ≥ 3, statistical analysis: paired t-test for whole curve or unpaired t-test for 96 h timepoint shown as (whole curve| 96 h) comparing monotreatment vs. combined treatment.

Supplementary Figure 6 WST assay comparing relative number of viable cells (viability; normalized to matched control) X vs. H of HT-1080, GCT, and RD cells following **(A)** radiation only with 4 and 8 Gy X (light/dark blue) or 4 and 8 Gy H (light/dark red) radiation. **(B-D)** combined treatment with **(B)** DoxA (before), **(C)** DoxB (before & after), and **(D)** DoxC (after). Dox treatment schedule details in . n ≥ 3, statistical analysis: paired t-test for whole curve or unpaired t-test for 96 h timepoint shown as (whole curve| 96 h) comparing matching X vs. H.

Supplementary Figure 7 WST assay analysing the additive effects of combined treatment compared with monotreatment (radiation only) of relative number of viable cells (viability; normalized to matched control) of HT-1080, GCT, and RD cells following combined treatment with 10 nM DoxA (before), DoxB (before & after), or DoxC (after) and **(A)** 4 Gy X (blue), **(B)** 8 Gy X (blue), **(C)** 4 Gy H (red), and **(D)** 8 Gy H (red). Dox treatment schedule details in . n ≥ 3, statistical analysis: paired t-test for whole curve or unpaired t-test for 96 h timepoint shown as (whole curve| 96 h) comparing monotreatment vs. combined treatment.

Supplementary Figure 8 Migration assay comparing relative number of motility X vs. H of HT-1080, GCT, and RD cells following **(A)** radiation only with 4 and 8 Gy X (light/dark blue) or 4 and 8 Gy H (light/dark red) radiation. **(B-D)** combined treatment with DoxA (before), **(C)** DoxB (before & after), and **(D)** DoxC (after) radiation. Dox treatment schedule details in . n ≥ 3, statistical analysis: paired t-test for whole curve comparing X vs. H (HT: 0-9h; GCT: 0-48h; RD: 0-48h).
