# Supplementary figures and images for: Similar additive effects of doxorubicin in combination with photon or proton irradiation in soft tissue sarcoma models
Source: Front Oncol. 2023 Jul 12;13:1211984. doi: 10.3389/fonc.2023.1211984 (PMC10368985; doi:10.3389/fonc.2023.1211984)

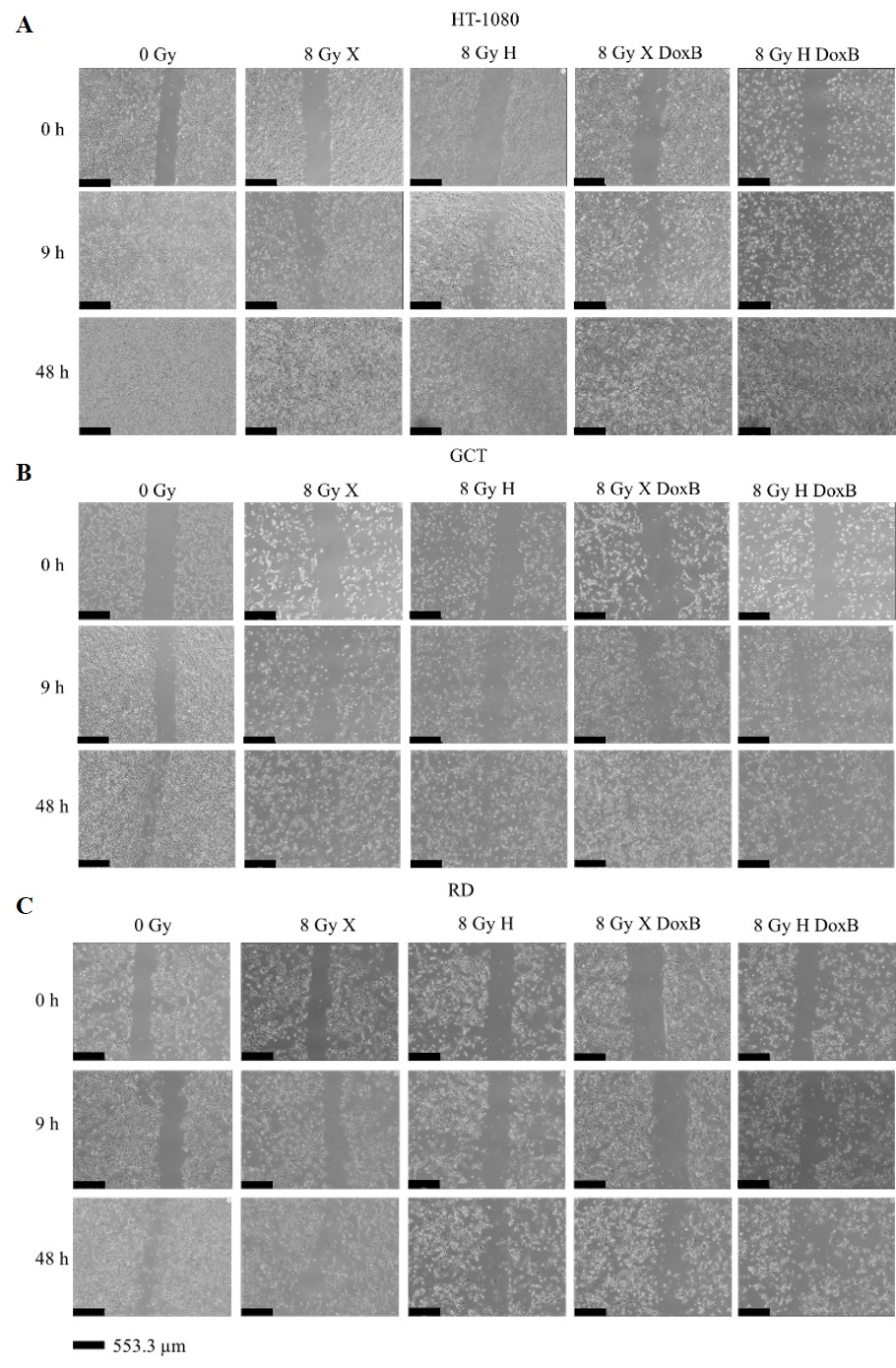

Supplement: Supplementary Figure 1 — Exemplary images of migration assay with (A) HT1080 cells, (B) GCT cells, and (C) RD cells following 8 Gy X or H radiation and combined treatment with DoxB (before & after; details see ) at different timepoints post scratch (0 h, 9 h, 48 h). 4 x magnification and scale bar = 553.3 µm. [file Image_1.jpg]

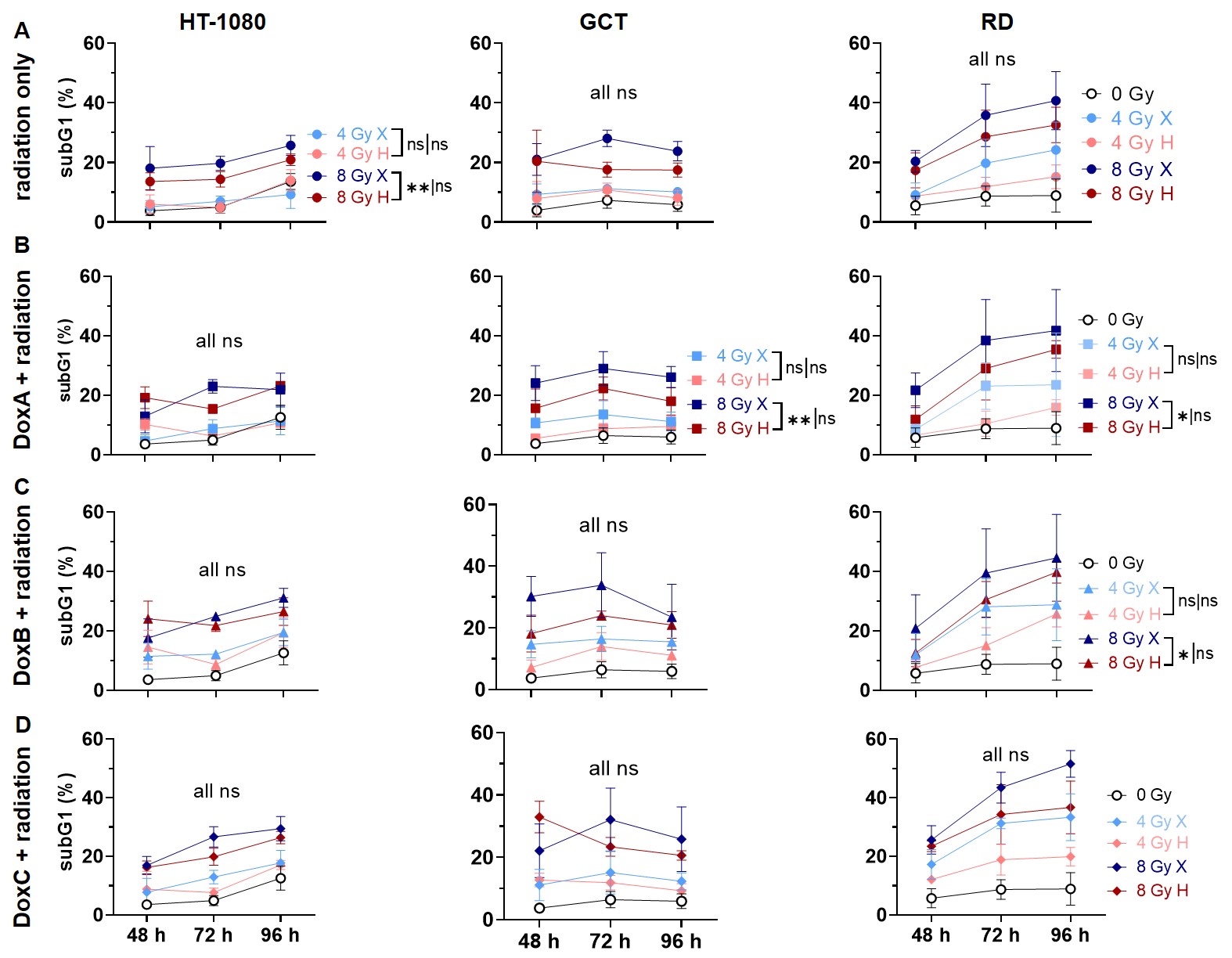

Supplement: Supplementary Figure 2 — Flow cytometry comparing relative subG1 phase X vs. H of HT-1080, GCT, and RD cells following (A) radiation only with 4 and 8 Gy X (light/dark blue) or 4 and 8 Gy H (light/dark red) radiation. (B-D) combined radiation treatment with (B) DoxA (before), (C) DoxB (before & after), and (D) DoxC (after). Dox treatment schedule details in . n ≥ 3, statistical analysis: paired t-test for whole curve or unpaired t-test for 96 h timepoint shown as (whole curve| 96 h) comparing matching X vs. H. [file Image_2.jpg]

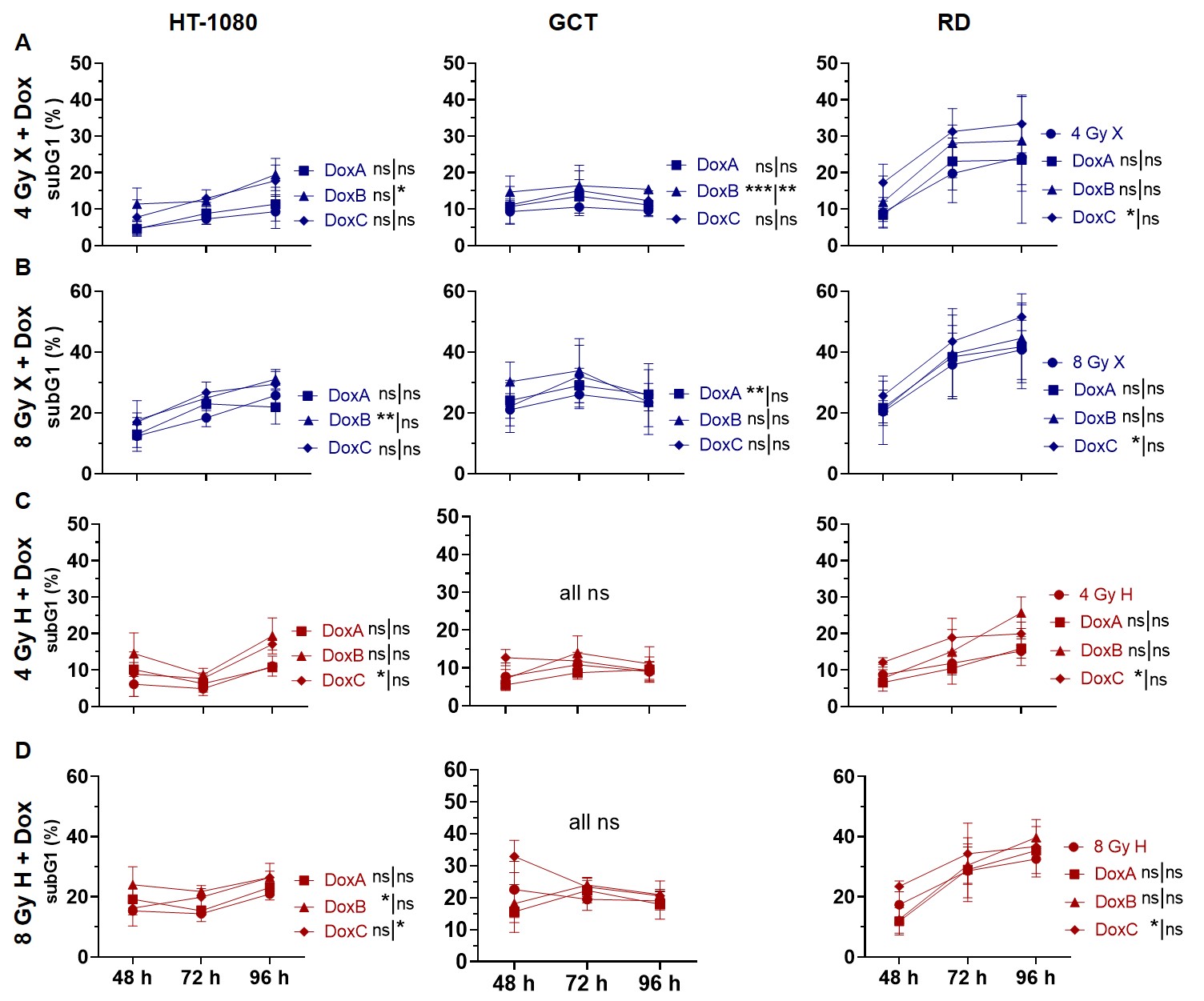

Supplement: Supplementary Figure 3 — Flow cytometry analysis of additive effects of combined treatment compared with monotreatment (radiation only) of relative subG1 phase (subG1) of HT-1080, GCT, and RD cells following combined treatment with 10 nM DoxA (before), DoxB (before & after), or DoxC (after) and (A) 4 Gy X (blue), (B) 8 Gy X (blue), (C) 4 Gy H (red), (D) 8 Gy H (red). Dox treatment schedule details in . n ≥ 3, statistical analysis: paired t-test for whole curve or unpaired t-test for 96 h timepoint shown as (whole curve| 96 h) comparing monotreatment vs. combined treatment. [file Image_3.jpg]

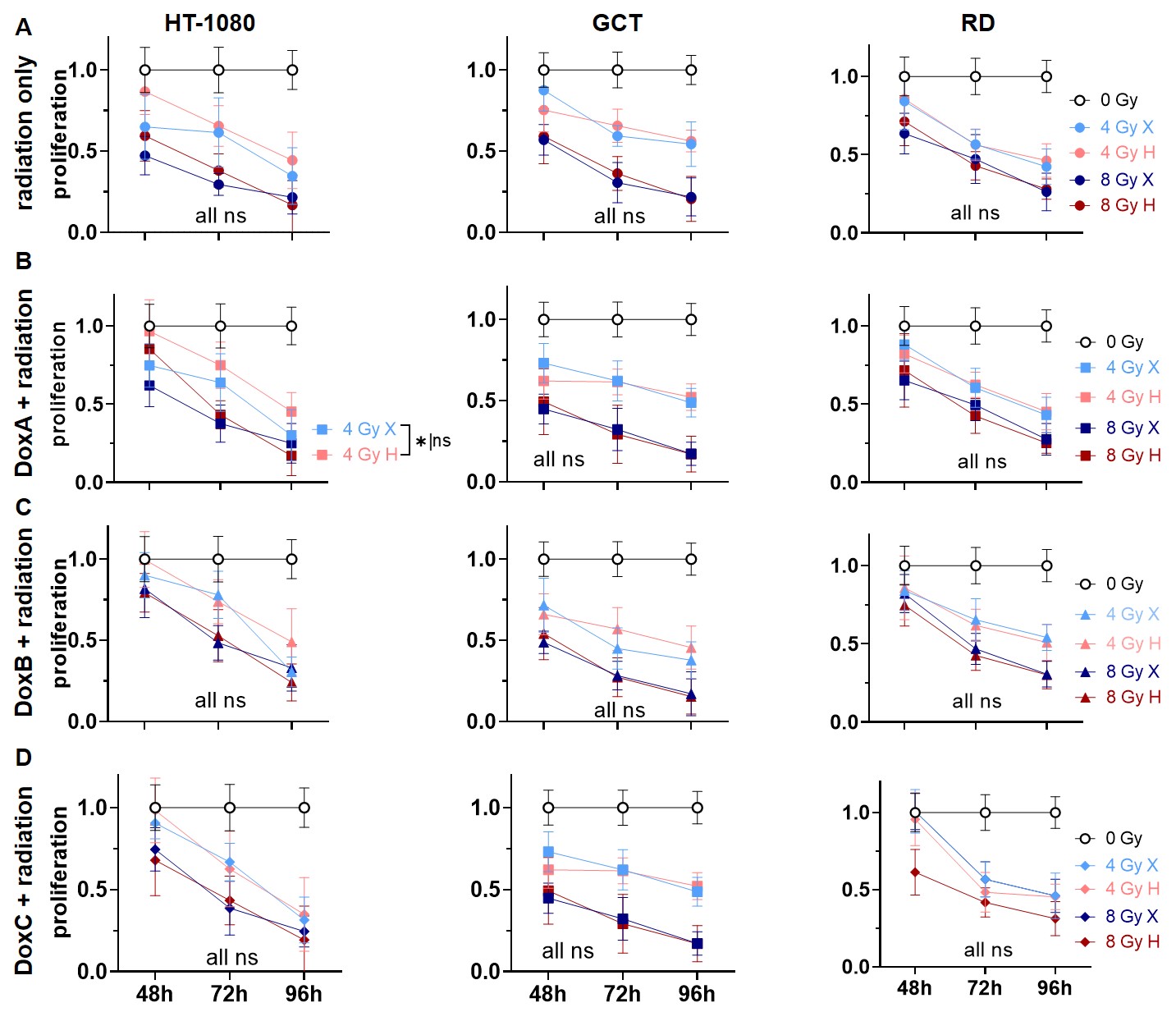

Supplement: Supplementary Figure 4 — CV assay comparing relative number of proliferating cells (proliferation; normalized to matched control) X vs. H of HT-1080, GCT, and RD cells following (A) radiation only with 4 and 8 Gy X (light/dark blue) or 4 and 8 Gy H (light/dark red) radiation. (B-D) combined radiation treatment with (B) DoxA (before), (C) DoxB (before & after), and (D) DoxC (after). Dox treatment schedule details in . n ≥ 3, statistical analysis: paired t-test for whole curve or unpaired t-test for 96 h timepoint shown as (whole curve| 96 h) comparing matching X vs. H. [file Image_4.jpg]

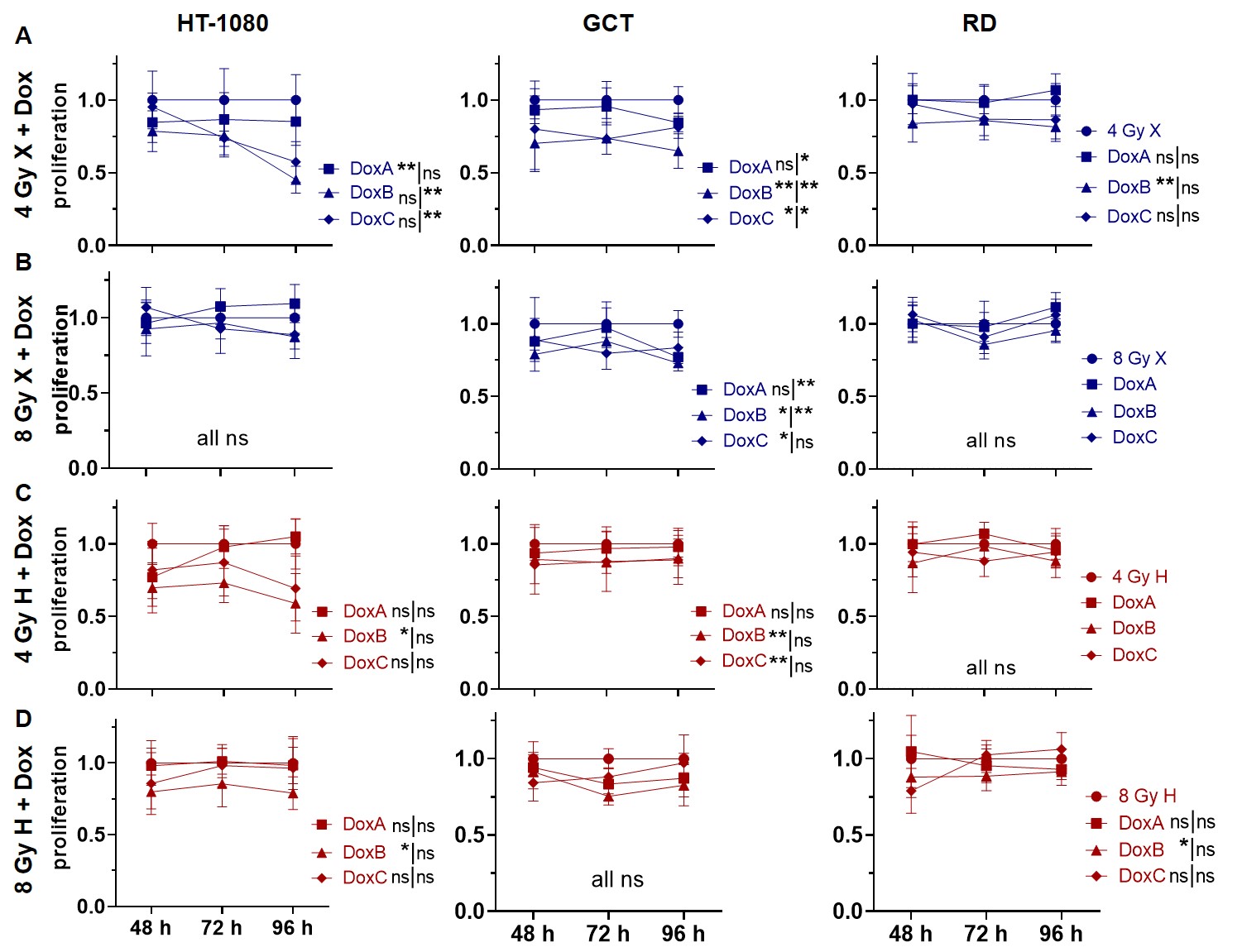

Supplement: Supplementary Figure 5 — CV assay analysing the additive effects of combined treatment compared with monotreatment (radiation only) of relative number of proliferating cells (proliferation; normalized to matched control) of HT-1080, GCT, and RD cells following combined treatment with 10 nM DoxA (before), DoxB (before & after) or DoxC (after) and (A) 4 Gy X (blue), (B) 8 Gy X (blue), (C) 4 Gy H (red), and (D) 8 Gy H.(red). Dox treatment schedule details in . n ≥ 3, statistical analysis: paired t-test for whole curve or unpaired t-test for 96 h timepoint shown as (whole curve| 96 h) comparing monotreatment vs. combined treatment. [file Image_5.jpg]

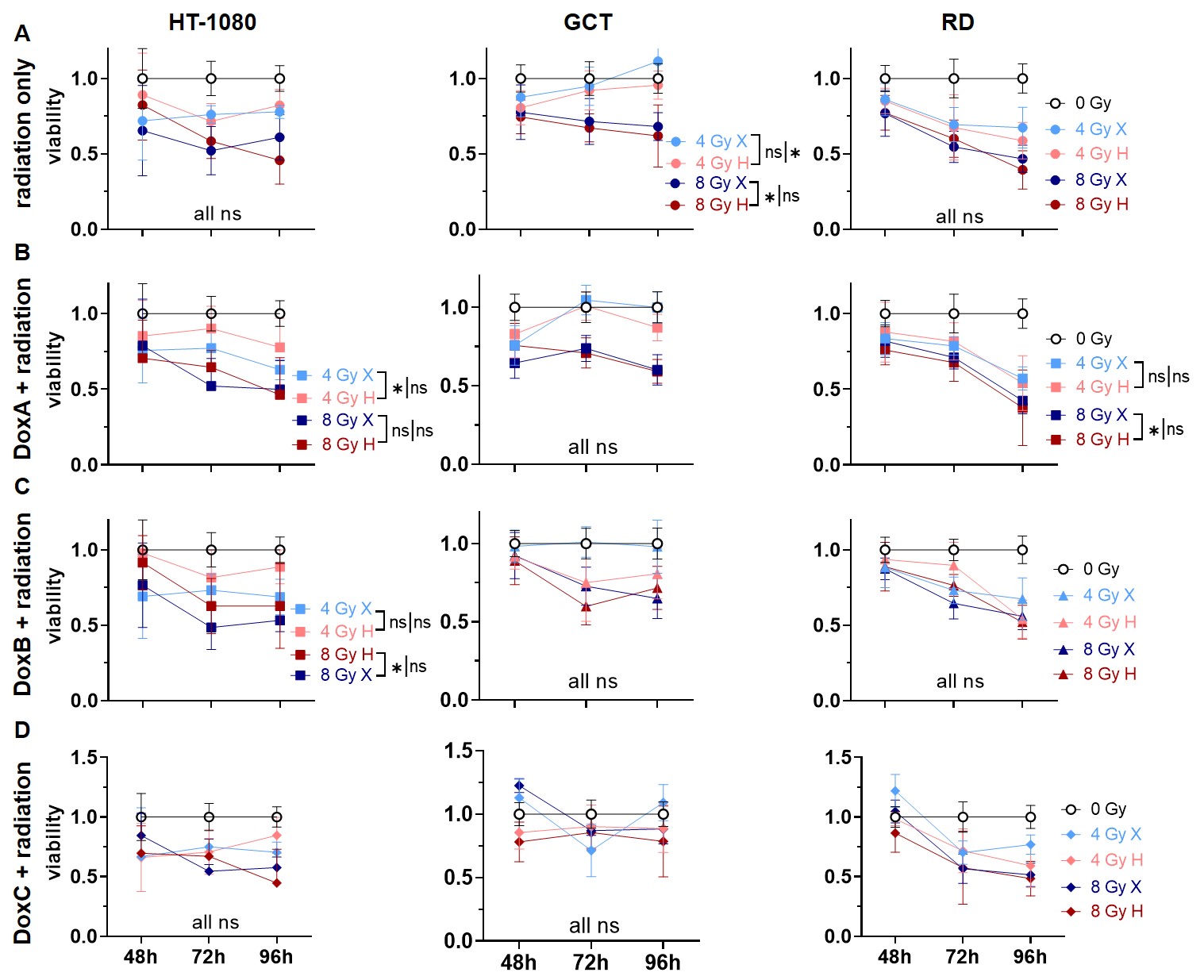

Supplement: Supplementary Figure 6 — WST assay comparing relative number of viable cells (viability; normalized to matched control) X vs. H of HT-1080, GCT, and RD cells following (A) radiation only with 4 and 8 Gy X (light/dark blue) or 4 and 8 Gy H (light/dark red) radiation. (B-D) combined treatment with (B) DoxA (before), (C) DoxB (before & after), and (D) DoxC (after). Dox treatment schedule details in . n ≥ 3, statistical analysis: paired t-test for whole curve or unpaired t-test for 96 h timepoint shown as (whole curve| 96 h) comparing matching X vs. H. [file Image_6.jpg]

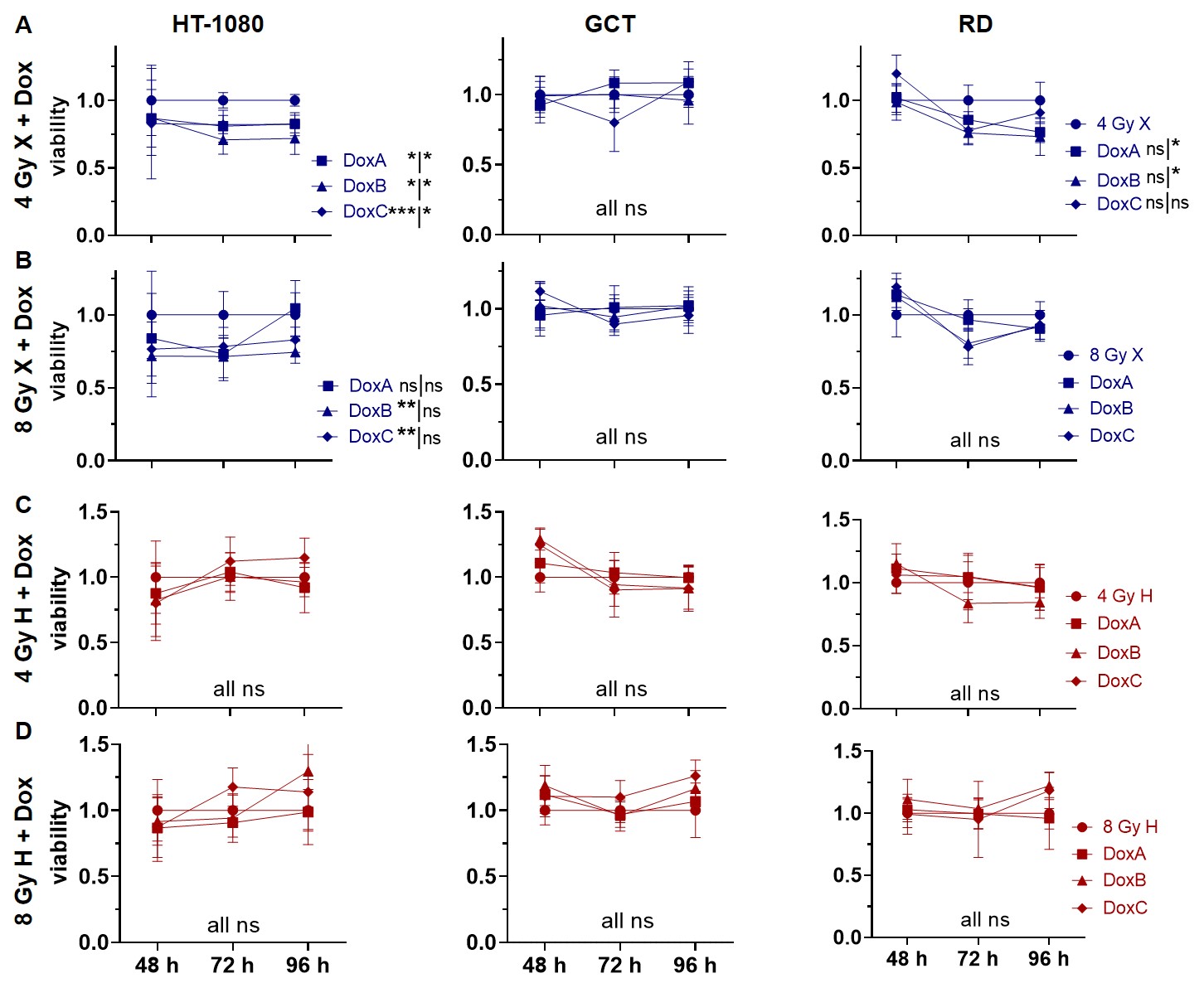

Supplement: Supplementary Figure 7 — WST assay analysing the additive effects of combined treatment compared with monotreatment (radiation only) of relative number of viable cells (viability; normalized to matched control) of HT-1080, GCT, and RD cells following combined treatment with 10 nM DoxA (before), DoxB (before & after), or DoxC (after) and (A) 4 Gy X (blue), (B) 8 Gy X (blue), (C) 4 Gy H (red), and (D) 8 Gy H (red). Dox treatment schedule details in . n ≥ 3, statistical analysis: paired t-test for whole curve or unpaired t-test for 96 h timepoint shown as (whole curve| 96 h) comparing monotreatment vs. combined treatment. [file Image_7.jpg]

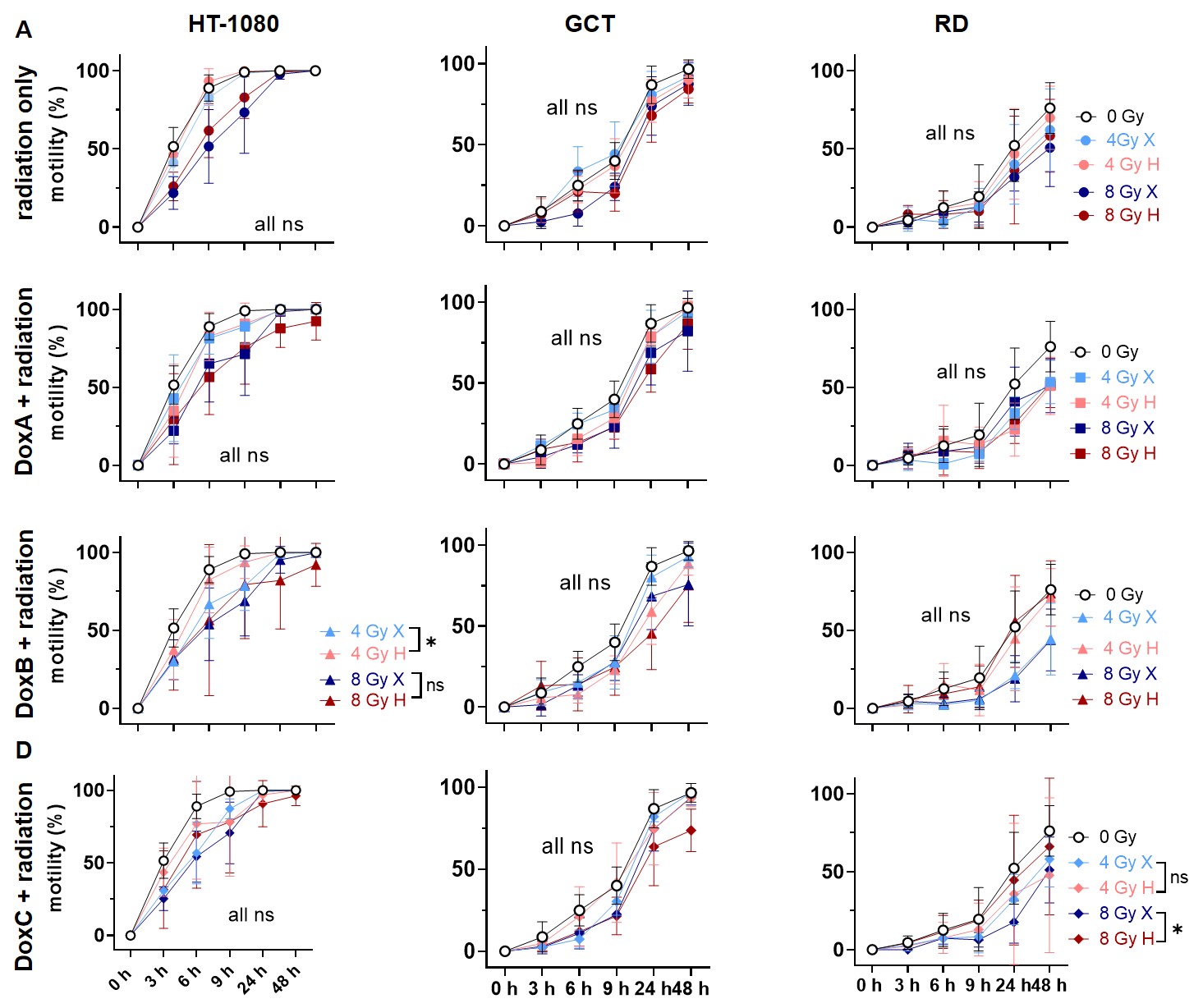

Supplement: Supplementary Figure 8 — Migration assay comparing relative number of motility X vs. H of HT-1080, GCT, and RD cells following (A) radiation only with 4 and 8 Gy X (light/dark blue) or 4 and 8 Gy H (light/dark red) radiation. (B-D) combined treatment with DoxA (before), (C) DoxB (before & after), and (D) DoxC (after) radiation. Dox treatment schedule details in . n ≥ 3, statistical analysis: paired t-test for whole curve comparing X vs. H (HT: 0-9h; GCT: 0-48h; RD: 0-48h). [file Image_8.jpg]

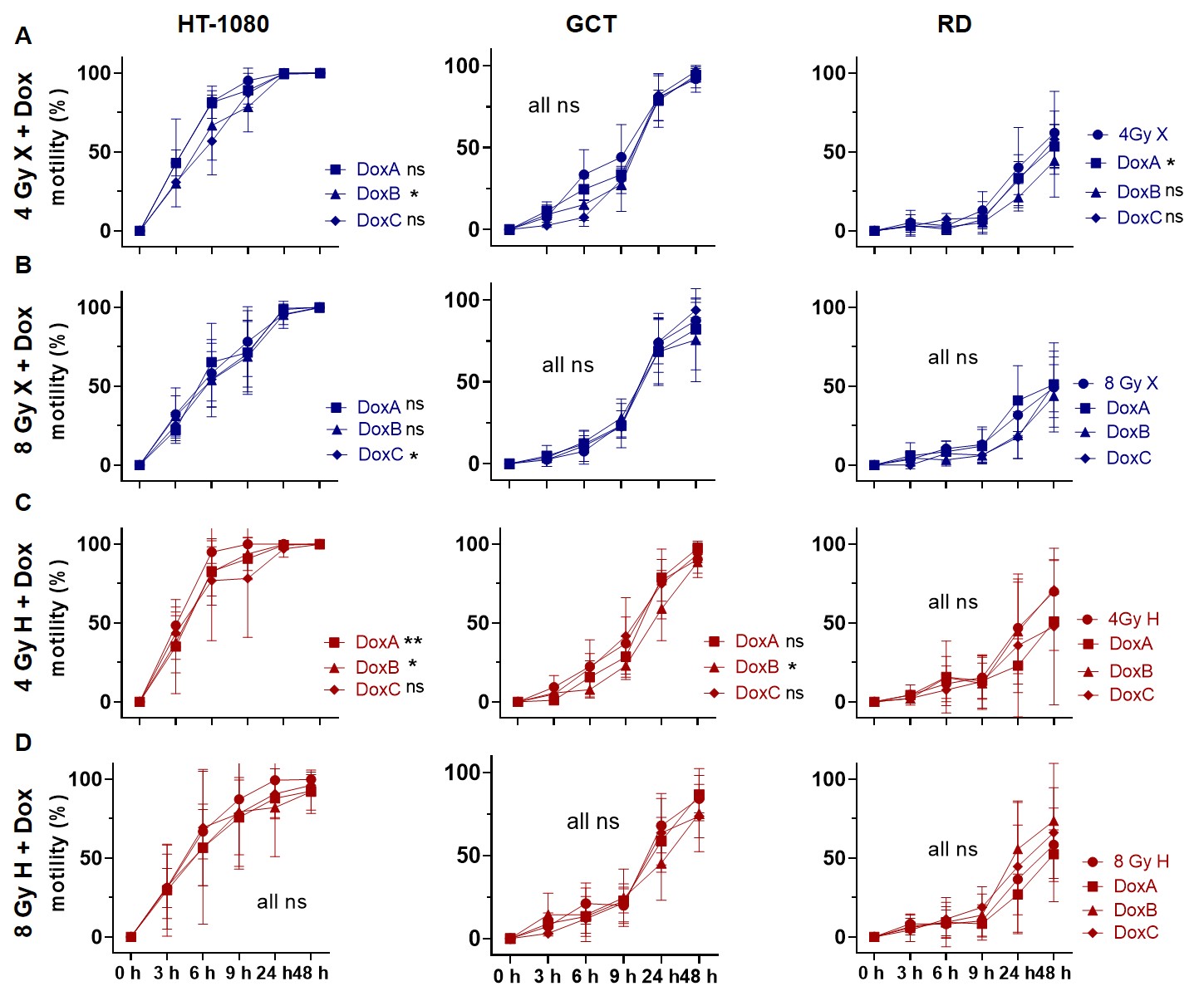

Supplement: Supplementary file 9 [file Image_9.jpg]
